# Previous Suture Type and Diameter of Fistula Predict Overall Repair Success for Post-Pneumonectomy Bronchopleural Fistulas

**DOI:** 10.5761/atcs.oa.25-00029

**Published:** 2025-07-10

**Authors:** Mustafa Akyıl, Volkan Baysungur

**Affiliations:** Department of Thoracic Surgery, Sureyyapasa Chest Diseases and Thoracic Surgery Training and Research Hospital, Istanbul, Turkey

**Keywords:** bronchopleural fistula, lung cancer, pneumonectomy

## Abstract

**Purpose:** This study aims to evaluate the treatment outcomes for patients who developed post-pneumonectomy bronchopleural fistula (BPF) and to identify factors that may influence the success of these treatment methods.

**Methods:** A cohort of 60 patients diagnosed with resistant BPF following pneumonectomy for non-small cell lung cancer was included in the study. Patients were categorized into 2 groups based on the efficacy of the BPF closure methods: successful closure and failed closure. Data on demographic, clinical, and pathological characteristics, surgical procedures, oncologic treatment status, laboratory parameters at the time of BPF diagnosis, fistula diameter, and bronchial stump length were collected. The effectiveness of bronchoscopic treatments and advanced surgical procedures was analyzed.

**Results:** Of the 60 patients included in the study, 55 (95%) were male, with a mean age of 61.6 ± 9.4 years. Multivariate analysis identified fistula diameter and the type of previous suture as significant predictors of BPF closure success ( *p* = 0.024 and 0.008, respectively).

**Conclusion:** Fistula diameter and previous suture type are critical determinants of the success of post-pneumonectomy BPF closure.

## Introduction

Post-pneumonectomy bronchopleural fistula (BPF) is one of the most severe and potentially life-threatening postoperative complications.^1,2)^ The incidence of post-pneumonectomy BPF in patients undergoing pneumonectomy for non-small cell lung cancer (NSCLC) has been reported to vary between 1.5% and 28%.^1,3–5)^

Several clinical studies over the years have identified surgical and patient-related factors contributing to the development of BPF.^3,6)^ However, a standardized treatment protocol for managing patients with BPF has yet to be established. Treatment options vary widely, from conservative approaches to more advanced surgical interventions. Key factors in determining the treatment strategy include the timing of BPF onset, the size of the fistula, the length of the bronchial stump or quality of residual stump tissue, the degree of cavity contamination, the overall condition of the patient, and the experience of the physician.^7)^

In cases of late or small BPF, conservative or bronchoscopic treatments are preferred. The most frequently employed bronchoscopic techniques include the application of fibrin glue, N-butyl cyanoacrylate, ethanol, and silver nitrate.^8,9)^ Conversely, surgical intervention may be warranted when the fistula diameter is large or when conservative measures fail. Surgical options include direct suturing, transsternal transpericardial access, contralateral lung intervention, muscle flap transposition, thoracoplasty, and omentoplasty.^10)^ This study aims to evaluate the outcomes of various treatment methods administered to patients with post-pneumonectomy BPF and to examine the factors that influence treatment success.

## Materials and Methods

**Fig. 1:**
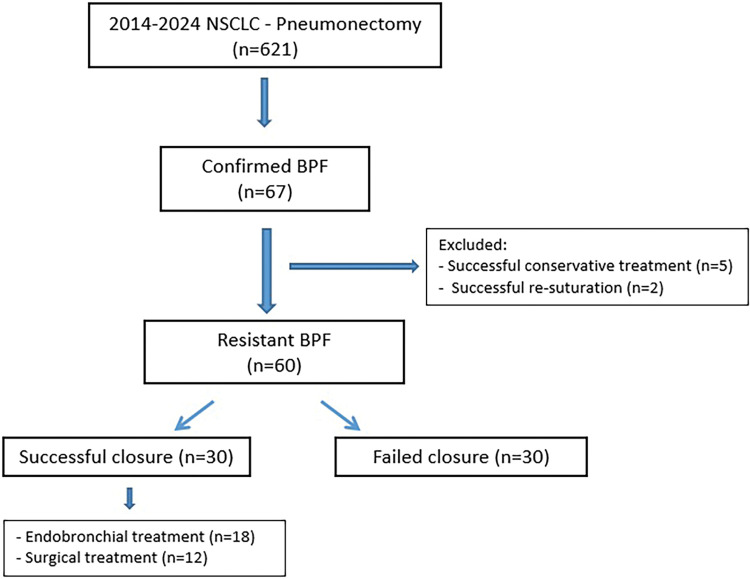
Flowchart of the patients. BPF: bronchopleural fistula; NSCLC: non-small cell lung cancer

This study is a retrospective, single-center observational analysis conducted at a teaching hospital designated as a reference center for chest diseases and thoracic surgery. Subjects were selected from interventional procedure notes and patient follow-up records, which are regularly reviewed by a thoracic surgery fellow in collaboration with a thoracic surgeon. The inclusion criteria comprised patients diagnosed with BPF who underwent pneumonectomy due to NSCLC between January 2014 and January 2024. Patient information was obtained from hospital and clinic electronic databases, surgical reports, patient files, and the Turkish national database. Patients who did not have regular follow-up visits or who experienced mortality during the early postoperative period were excluded from the study (**Fig. 1**).

### Hospital and clinic organization

Our thoracic surgery clinic operates with 40 inpatient beds and 10 postoperative intensive care unit beds, facilitating surgeries across 3 operating tables. It serves as a referral center for patients from throughout Turkey, particularly from Istanbul. During the study period, decisions regarding surgical interventions and treatment methods were determined in surgical councils. Post-discharge follow-up care was similarly outlined during these councils to ensure that all patients, including those with early-stage lung cancer, were referred to the medical oncology department for ongoing outpatient care in our clinics.

### Patient management

Pneumonectomy was performed in patients whose preoperative forced expiratory volume in 1 second (FEV1) exceeded 2 L or 80%. For those presenting with suboptimal pulmonary function test results, additional assessments were performed, including quantitative perfusion scintigraphy, maximal oxygen consumption (VO_2_max), and the 6-minute walk test. An adequate postoperative FEV1 value was defined as ≥800 mL or 40% of the predicted value.^11)^ During the procedures, all patients were monitored under standard double-lumen intubation by an anesthesiologist. Bronchial closure was performed using an automatic bronchial stapler (TA 30-4.8, Autosuture; US Surgical, Norwalk, CT, USA), 3/0 monofilament nonabsorbable polypropylene sutures (Prolene; Ethicon, Inc., Somerville, NJ, USA), and 3/0 polyglactin sutures (Vicryl; Ethicon, Inc., Somerville, NJ, USA). After bronchial stump closure, air leak control was performed with 30–40 cm H_2_O pressure. Based on our institutional approach and experience, in patients with elevated intraoperative risk of bronchial stump dehiscence, we preferred to reinforce the stump using intrathoracic tissues, such as pericardial fat pads or pleural flaps, which offer mechanical support without the need for additional abdominal intervention. Following mediastinal lymph node dissection, a 32-French polyethylene chest tube was placed, and the procedure was completed. Patients were monitored in the intensive care unit for 24 hours postoperatively, with underwater-sealed tube thoracostomy discontinued within the first 48 hours.

### BPF diagnosis and treatment approach

Diagnostic timing is important in determining treatment plans for patients with BPF. Patients diagnosed within the first 30 days post-surgery were classified as “Early BPF,” while those diagnosed after this period were classified as “Late BPF.”^12,13)^ For patients with early BPF, re-suturing was performed via thoracotomy, provided the patient’s condition was stable. Late BPF patients were diagnosed either in our emergency department, during outpatient appointments, or in external facilities and subsequently referred to our center. Clinical presentation most commonly included cough, dyspnea, and frequent expectoration. The observation of reduced fluid levels or air–fluid levels on chest X-rays was also common. Diagnosis was confirmed with fiberoptic bronchoscopy in all patients. As part of conservative treatment, for patients with underwater-sealed tube thoracostomy, pleural irrigation was performed using appropriate antibiotics and antiseptic solutions, along with administration of albumin and protein supplementation. The primary aim of this treatment approach was to control infection and prevent the development of contralateral pneumonia, which is a critical aspect in managing bronchial stump fistulas. By ensuring effective drainage and appropriate antimicrobial therapy, the risk of secondary infections, including contralateral pneumonia, was minimized, thereby improving overall outcomes.^14)^

Conservative treatment was applied for 1 month, after which fiberoptic bronchoscopy was conducted for patients with resolved air leaks. If patients no longer had evidence of BPF, the chest tubes were removed. Patients with unsatisfactory treatment outcomes were referred to the surgical council once infection parameters were controlled.

Patients in whom closure of the BPF failed despite surgical intervention for early BPF and those in whom BPF did not close despite conservative management for late BPF were classified as having “Resistant BPF.”

Initially, in patients with stable general conditions, bronchial closure was performed using rigid bronchoscopy with N-butyl cyanoacrylate. In cases with successful air leak cessation, chest tube removal was confirmed by fiberoptic bronchoscopy and subsequently terminated. No chest tube was removed before 30 days post-surgery.

The surgical council evaluated advanced surgical options for all patients who experienced a failure of bronchoscopic treatment. Patients deemed medically inoperable were managed with chronic tube thoracostomy and scheduled outpatient visits. Operable patients underwent individualized surgical procedures, including omentoplasty, muscle flap transposition, and re-stapling of the left main bronchial stump through a right thoracotomy approach. Following the 30-day postoperative period, fiberoptic bronchoscopy was performed, and chest tubes were removed in patients without BPF. Regular follow-up protocols included monitoring for pleural fluid accumulation and clinical status through posteroanterior chest radiographs. In instances where surgical closure remained unsuccessful, management via chronic tube thoracostomy continued.

### Recorded parameters

Demographic and clinical data for each patient were obtained from medical files. This included comprehensive documentation of comorbidities, smoking status, smoking load (packs/year), lesion location, and date(s) of the surgical operation for pneumonectomy and BPF closure as applicable. Additional details concerning the type of pneumonectomy performed, tumor histology, bronchial margin positivity of the resected material, methods of bronchial closure, time until chest tube removal, and duration of hospital stay were also recorded. Moreover, the tumor-node-metastasis (TNM) stage of malignancy was classified according to the 8th edition of the TNM classification system and recorded, along with information regarding the status of neoadjuvant and adjuvant oncologic treatments.^15)^

At the time of BPF diagnosis, laboratory parameters were documented for each patient. These included leukocyte count (10^3^/L), neutrophil count (10^3^/L), lymphocyte count (10^3^/L), platelet count (10^3^/L), mean corpuscular volume in femtoliters (fL), mean platelet volume in femtoliters (fL), C-reactive protein (mg/dL), hemoglobin (g/dL), hematocrit as a percentage (%), blood urea nitrogen (mg/dL), creatinine (mg/dL), albumin (g/dL), sodium (mmol/L), and potassium (mmol/L).

Computed tomography, fiberoptic bronchoscopy, and rigid bronchoscopy were employed to estimate the diameter of the BPF and the length of the bronchial stump. Treatment methods and outcomes were recorded. Patients were divided into 2 groups based on the success of the BPF closure methods: successful closure (Group 1) and failed closure (Group 2). A comparative analysis of demographic, laboratory, and clinical characteristics was conducted between these 2 groups, and the effectiveness of bronchoscopic interventions and advanced surgical procedures was investigated.

### Statistical analysis

Quantitative data are expressed as mean ± standard deviation (SD), and qualitative data are expressed as frequencies. Student’s t-test and chi-square tests were used to evaluate potential factors associated with the success of BPF closure. Logistic regression analysis was performed to identify independent predictors of procedural success. Variables that exhibited a significance level of *p* <0.1 in univariate analyses were subjected to further evaluation via logistic regression. All statistical analyses were conducted using the statistical software package SPSS for Windows, version 16.0 (SPSS Inc., Chicago, IL, USA). A *p*-value of less than 0.05 was considered significant.

## Results

Of the 60 patients in this study, 55 (95%) were male, with a mean age of 61.6 ± 9.4 years. Twenty-nine patients (48%) underwent right pneumonectomy, while 31 (52%) underwent left pneumonectomy. Histopathological diagnosis revealed squamous cell carcinoma in 45 patients and adenocarcinoma in 15 patients. A total of 40 patients received adjuvant therapy (**Table 1**).

**Table 1:** Comparison of BPF patients with successful closure and failed closure

	Successful closure (N = 30, %)	Failed closure (N = 30, %)	*p*
Age (year)	61.2 ± 10.7	62 ± 8	0.745
Gender			
Male	27 (90)	28 (93)	0.999
Female	3 (10)	2 (7)	
Smoker	29 (97)	28 (93)	0.875
Smoking load (pack/year)	35.5 ± 18	37.8 ± 16	0.617
Any comorbidities	20 (67)	24 (80)	0.382
Comorbidities			
COPD	12 (40)	12 (40)	0.999
HT	6 (20)	8 (26)	0.476
DM	5 (17)	7 (23)	0.748
CAD	4 (13)	7 (23)	0.506
TB	4 (13)	4 (13)	0.999
Neoadjuvan treatment	2 (6)	3 (10)	0.999
Side of pneumonectomy			
Right	16 (54)	13 (43)	0.606
Left	14 (46)	17 (57)	
Pneumonectomy type			
Standard	21 (70)	17 (57)	
Intrapericardiac	9 (30)	10 (33)	0.176
Completion	0	3 (10)	
Bronchus closure			
Stapler	25 (83)	19 (63)	**0.041**
Prolen	1 (4)	8 (27)	
Vicryl	4 (13)	3 (10)	
Bronchial margin positive	3 (10)	4 (13)	0.999
Duration to removal of the chest tube (days)	1.7 ± 1	1.83 ± 0.8	0.589
Hospital stay (days)	8.5 ± 5.5	6.8 ± 2.3	0.675
Tumor histology			
SCC	25 (83)	20 (66)	**0.024**
Adenocarcinoma	5 (17)	10 (34)	
Tumor length (average mm)	45.8 ± 17.4	44.6 ± 2.3	0.821
Pathological stage			
IIIB	3 (10)	2 (7)	
IIIA	8 (27)	10 (33)	0.453
IIB	13 (43)	10 (33)	
IIA	1 (3)	4 (13)	
IB	3 (10)	3 (10)	
IA	2 (7)	1 (3)	
N factor			
N0	10 (33)	14 (46)	0.550
N1	16 (53)	12 (40)	
N2	4 (14)	4 (14)	
Adjuvant treatment			
None	8 (26)	12 (43)	
Chemotherapy	11 (37)	4 (11)	0.158
Chemoradiotherapy	11 (37)	14 (46)	
Length of the bronchial stump (cm)	1.8 ± 0.3	2 ± 0.6	0.204
BPF (diameter mm)	4.3 ± 3.4	5.8 ± 2.9	**0.048**

Bold numerals indicate statistically significant results (*p* <0.05).

CAD: coronary artery disease; COPD: chronic obstructive pulmonary disease; DM: diabetes mellitus; HT: hypertension; SCC: squamous cell carcinoma; TB: tuberculosis

All 60 patients in this study were diagnosed with resistant BPF and were treated with N-butyl cyanoacrylate via rigid bronchoscopy. Endobronchial treatment was successful in 18 patients (30%). When comparing the group in which endobronchial treatment was successful with the group in which it failed, the diameter of the BPF was found to be significantly larger in the failed closure group (2.68 ± 1.54 vs. 5.79 ± 2.83 mm, *p* <0.001). In terms of pneumonectomy type, the success rates of endobronchial closure were 48% for standard pneumonectomy, 20% for intrapericardial pneumonectomy, and 0% for completion pneumonectomy, with standard pneumonectomy showing a statistically significant association with better outcomes (*p* = 0.031). Furthermore, squamous cell carcinoma histology was identified as an independent predictor of successful endobronchial treatment (*p* = 0.032).

Of the other 42 patients (70%), for whom endobronchial treatment was unsuccessful, 21 were considered medically inoperable. The remaining 21 patients underwent advanced surgical procedures. These procedures included omentoplasty in 13 patients, serratus muscle flap in 4 patients, and contralateral thoracotomy in 4 patients. Surgical intervention successfully closed the BPF in 12 cases (57%), with omentoplasty demonstrating the highest success rate (61.5%) (**Table 2**). Thus, the successful closure group (Group 1) was composed of the 18 patients for whom endobronchial treatment was successful, in addition to the 12 patients whose BPFs were successfully closed via advanced surgical methods. A total of 30 patients, who were either classified as medically inoperable or experienced surgical treatment failure, formed the failed closure group (Group 2) and were managed with chronic tube thoracostomy.

**Table 2:** Treatment methods used for patients with a post-pneumonectomy bronchopleural fistula

Treatment method	N	Successful closure	Failed closure	Success of treatment (%)	*p*
Endobronchial treatment					
N-butyl cyanoacrylate	60	18	42	30	0.552
Surgical treatment	21	12	9	57.1	
Omentoplasty	13	8	5	61.5	0.999
Serratus muscle flap	4	2	2	50	
Contralateral thoracotomy	4	2	2	50	

A comparison of the successful closure group (Group 1) and the failed closure group (Group 2) revealed no statistically significant differences in mean age, gender distribution, smoking status, comorbidities, or neoadjuvant therapy status (*p* >0.05). Factors such as the side of pneumonectomy, type of surgical procedure, time until chest tube removal, and duration of hospital stay did not significantly impact BPF closure success (*p* >0.05). Notably, when evaluating bronchial closure methods used during pneumonectomy, patients whose bronchial stump was closed with Prolene exhibited a significantly lower success rate in BPF closure compared to those with stapler or Vicryl closure (*p* = 0.041) (**Table 1**).

In univariate analysis, squamous cell carcinoma histology emerged as a significant predictor of BPF closure success (*p* = 0.024). Conversely, bronchial margin positivity, tumor length, pathological stage, N factor, and adjuvant treatment status did not exhibit significant effects on BPF closure outcomes (*p* >0.05). While the length of the bronchial stump was not significantly different between the 2 groups (1.8 ± 0.3 vs. 2 ± 0.6 mm, *p* = 0.204), the diameter of the BPF tended to be significantly larger in the failed closure group (4.3 ± 3.4 vs. 5.8 ± 2.9 mm, *p* = 0.048; **Table 1**). Univariate analysis did not reveal any statistically significant differences between Group 1 and Group 2 regarding the laboratory parameters recorded at the time of BPF diagnosis (*p* >0.05 for all parameters; **Table 3**).

**Table 3:** Comparison of laboratory parameters of patients at the time of BPF diagnosis between 2 groups

	Successful closure (mean ± SD)	Failed closure (mean ± SD)	*p*
CRP (mg/dL)	119.3 ± 80	106.5 ± 70.6	0.611
WBC (10^3^/μL)	10.3 ± 4.1	10.1 ± 3.9	0.863
Neutrophil (10^3^/μL)	7.5 ± 3.2	7.2 ± 3.2	0.756
Lymphocyte (10^3^/μL)	1.4 ± 0.5	1.8 ± 0.7	0.530
MPV (fL)	8.2 ± 0.9	8.2 ± 1.5	0.792
MCV (fL)	83.9 ± 5.6	82.3 ± 7.3	0.328
Hgb (g/dL)	10.9 ± 1.3	10.7 ± 1.1	0.624
HCT (%)	33.6 ± 3.7	33.3 ± 3.4	0.784
Platelet (10^3^/μL)	402 ± 139	410 ± 164	0.828
BUN (mg/dL)	33.8 ± 10.7	34.7 ± 10.9	0.740
Creatinine (mg/dL)	0.79 ± 0.21	0.75 ± 0.17	0.355
Albumin (g/dL)	3.3 ± 0.6	3.4 ± 0.5	0.555
Sodium (mmol/L)	138 ± 2.6	136.9 ± 3	0.119
Potassium (mmol/L)	4.4 ± 0.4	4.5 ± 0.5	0.525

BUN: blood urea nitrogen; CRP: C-reactive protein; HCT: hematocrit; Hgb: hemoglobin; MCV: mean corpuscular volume; MPV: mean platelet volume; WBC: white blood cell count

In multivariate analysis, parameters previously deemed significant in the univariate analysis were further evaluated. The diameter of the fistula and previous suture type (Prolene) were identified as independent factors influencing the success of BPF closure treatment (*p* = 0.024, *p* = 0.008; **Table 4**).

**Table 4:** Logistic regression analysis results

	Coefficient	SE coefficient	Z	*p*
Tumor histology (SCC)	0.256	0.148	1.729	0.090
BPF (mm)	0.042	0.018	2.333	**0.024**
Bronchus closure (Prolene)	0.429	0.154	2.789	**0.008**

Bold numerals indicate statistically significant results (*p* <0.05).

SCC: squamous cell carcinoma; BPF: bronchopleural fistula

The mean overall survival rates were comparable between the successful closure group (62.6 ± 36 months) and the failed closure group (51.7 ± 39 months), with a *p*-value of 0.275. Mortality occurred in 16 patients (27%), 15 of whom were in the failed closure group. This subgroup had a mean survival time of 16.6 ± 4.8 months, with a range extending from 1 to 68 months and a median survival time of 17.5 months.

## Discussion

Pneumonectomy is associated with higher morbidity and mortality rates than all other surgical interventions for lung cancer.^1–3)^ One of the most serious and potentially fatal postoperative complications is the development of a BPF.^1,2)^ The reported incidence of BPF in patients who have undergone pneumonectomy for NSCLC ranges from 1.5% to 28%.^3–5)^ The occurrence of BPF is higher following right pneumonectomy compared to left pneumonectomy, a disparity largely attributed to anatomical differences.^1,6,16)^ The right main bronchus is wider and has a more vertical angle, leading to greater secretion accumulation.^2)^ Moreover, the right main bronchus is vascularized by a single bronchial artery and is covered by less mediastinal tissue.^17)^

Several surgical factors contribute to the risk of developing BPF. These factors include the techniques employed for bronchial closure, the presence of tumors at the bronchial surgical margin, the length of the bronchial stump, excessive lymph node dissection, completion pneumonectomy, and the surgeon’s experience. Additionally, patient-related factors such as nutritional status, hypoalbuminemia, neoadjuvant therapy, diabetes mellitus, tuberculosis, steroid use, preoperative or postoperative pulmonary infections, as well as prolonged postoperative mechanical ventilation, can also influence outcomes.^1,2,6)^

Currently, there is no established protocol for managing BPF in affected patients. Treatment options vary from conservative approaches to more complex surgical procedures. The choice of treatment is contingent upon several factors, including the timing of BPF occurrence, its size, the length of the bronchial stump, the quality of remaining stump tissue, contamination of the thoracic cavity, the overall health of the patient, and the experience of the physician.^7)^ Initial treatment involves pleural cavity drainage, lavage with sterile solutions, and appropriate antibiotic therapy. A subsequent bronchoscopic assessment of the BPF is then indicated.^18)^

Early BPF was defined as fistulas occurring within 30 days after pneumonectomy, while late BPF referred to fistulas occurring beyond this period. This classification is widely used in the literature, although slight variations exist. For example, Okuda et al. and Mazzella et al. adopted the 30-day division,^12,13)^ whereas Varoli et al. proposed a 3-stage timing-based classification (early: 1–7 days; intermediate: 8–30 days; late: >30 days).^19)^ In light of this literature and these studies' practical implications, we also employed the postoperative 30-day-limit threshold for BPF formation timing. Due to the limited number of early BPF cases and the convergence of treatment strategy in resistant cases, both early and late BPFs were analyzed together. Early postoperative BPFs, which occur within the 1st week, may arise due to local infection, ischemia, or technical complications.^2)^ In cases of early BPF presenting significant clinical symptoms, re-thoracotomy and surgical repair are preferred.^1)^ In most cases, inflammation prevents the use of staplers, necessitating the closure of the stump with single monofilament sutures after mobilizing the bronchial stump.^20)^ Furthermore, supporting the healing of the bronchial stump through the use of well-perfused tissues such as intercostal muscle flaps, parietal pleura, or pericardial fat pads has shown promise in facilitating recovery.^21)^ In our study, re-suturing was performed in 3 patients with early fistulas, resulting in successful closure for 2 of these cases.

In cases of late and minimal BPFs, conservative or bronchoscopic intervention is preferred. The size, location, and shape of the fistula all influence the success of bronchoscopic treatment. Moreover, there are risks associated with bronchoscopic treatment, such as potential enlargement of the fistula secondary to infection and aspiration of closure materials into the contralateral lung.^22)^ The advantage of bronchoscopic closure in selected cases is that no surgical burden is required. Commonly used methods in bronchoscopic treatment include fibrin glue, N-butyl cyanoacrylate, ethanol, and silver nitrate.^8,9)^ Boudaya et al. describe favorable outcomes with silver nitrate application via bronchoscopy.^8)^ Chawla et al. report an 88.8% success rate in 9 patients who underwent bronchoscopic N-butyl cyanoacrylate application for BPF.^23)^ In our study, successful closure was achieved in 18 (30%) patients using bronchoscopic N-butyl cyanoacrylate. Another bronchoscopic technique involves using metallic silicone-coated stents and silicone-modified Y stents. Han et al. noted a success rate of 96.6% with customized airway stents.^24)^ In a retrospective analysis of 35 patients with pneumonectomy-related BPFs undergoing bronchoscopic repair, Cardillo et al. showed that patients with BPFs with fistula diameters of ≤2 mm had a success rate of 92.3%, while those with fistulas >6 mm had only a 33.3% success rate.^25)^ Approaches utilizing tissue adhesives exhibit diminishing success rates for fistulas >8 mm and are generally regarded as inappropriate in such cases.^26)^ In our study, the BPF diameter was significantly larger in the failed closure group than in the successful closure group (4.3 ± 3.4 vs. 5.8 ± 2.9 mm, *p* = 0.048).

Surgical intervention may be required when the BPF diameter is large or does not resolve with conservative treatment. Surgical options include re-suturing, transsternal transpericardial approaches, contralateral thoracotomy, muscle flap transposition, thoracoplasty, and omentoplasty.^10)^ The most commonly used muscle flaps include the latissimus dorsi, pectoralis major, serratus anterior, pectoralis minor, and rectus abdominis muscles.^27)^ In our study, among the patients who underwent omentoplasty (n = 13), the observed success rate was 61.5%. However, due to the limited sample size, a meaningful subgroup analysis to compare clinical or procedural characteristics between successful and unsuccessful cases could not be performed. This limitation should be considered when interpreting the clinical utility of omentoplasty in this setting. Further studies with larger patient cohorts are needed to better define the factors influencing the success of this surgical approach.

All omentoplasty procedures analyzed in our cohort were performed after the diagnosis of BPF. Although some centers have advocated the use of prophylactic omental flaps in high-risk patients, as reported by Grunenwald et al. ^28)^ in cases involving thoracic radiotherapy and pneumonectomy, this practice has not been adopted routinely in our center. One of the major reasons is that omentoplasty requires laparotomy and access to the abdominal cavity, which increases operative time and carries additional risks such as intra-abdominal infection, hernia, or organ injury.

Another surgical approach to BPF involves closing the fistula using a contralateral thoracotomy approach. This method achieves closure with either a stapler or nonabsorbable sutures, positioned just distal to the carina and proximal to the bronchial stump, without entering the fistulized hemithorax.^29)^ In instances of late post-pneumonectomy BPF, re-thoracotomy for fistula closure presents notable challenges, chiefly the potential for fibrothorax development and the risk of injury to the pulmonary artery stump. Extracorporeal membrane oxygenation (ECMO) support may help mitigate some risks in such situations. For instance, Li et al. reported that ECMO support provided oxygenation assurance and time for treatment in cases of left BPF via a right thoracic approach.^29)^ Moreno et al. documented a 100% success rate in a series of 5 patients who underwent a contralateral thoracotomy approach.^30)^ In contrast, our study yielded a success rate of 50% among 4 patients treated via a contralateral thoracotomy approach.

Zhu et al. evaluated 47 BPF patients with chronic post-pneumonectomy empyema using 3-dimensional reconstruction computed tomography scans and performed surgical treatment with regional muscle flaps. They reported a 93.6% success rate across 44 patients, with the most commonly used muscle flap being the latissimus dorsi.^31)^ In our study, we applied serratus muscle flaps in 4 patients, with a 50% success rate. In cases where surgical interventions fail, thoracoplasty may be considered. This technique involves resection of rib portions to facilitate chest wall collapse. While thoracoplasty carries a high mortality rate, it offers the advantage of enabling more effective infection control by eliminating the pleural cavity.^32)^

Several studies have analyzed the association between bronchial closure techniques and the development of BPF. Hubaut et al. reported a lower incidence of BPF in a cohort of 209 pneumonectomies when manual suturing was employed, as opposed to stapling.^33)^ However, Skrzypczak et al. found no significant discrepancy in BPF occurrence between the stapler and manual suturing methods in their analysis of 455 pneumonectomy patients (*p* = 0.72).^16)^ To our knowledge, no existing literature has specifically investigated the impact of the type of suture used during pneumonectomy on BPF treatment outcomes. In our study, patients whose bronchial stump was closed with Prolene had a significantly lower success rate for BPF closure (only 1 out of 9 patients) in comparison to those closed with stapler or Vicryl closure (*p* = 0.041).

Though this study has limitations inherent to its retrospective, single-center design, the observed incidence of post-pneumonectomy BPF aligns with existing literature. The number of patients who underwent advanced surgical procedures is relatively small. Nevertheless, a notable strength of this study lies in its execution within a specialized thoracic surgical clinic at a dedicated hospital, which allowed for comprehensive patient follow-up and provided a sufficiently large patient population for statistical analysis.

## Conclusion

There is a scarcity of research examining the effectiveness of BPF treatments, with existing studies often characterized by insufficient sample sizes to establish a clear protocol. The selection of treatment method is largely influenced by the experience of the center and the discretion of the surgeon. It is imperative to consider surgical and patient-specific risk factors to ensure that appropriate treatment modalities are employed when BPF develops. Future research should focus on identifying predictive factors for resistance to treatment in BPF, as well as exploring alternative therapeutic approaches that may improve outcomes for patients with resistant BPF.

## Declarations

### Ethics approval and consent to participate

The study protocol was approved by the Süreyyapaşa Chest Disease and Thoracic Surgery Training and Research Hospital Ethics Committee (2024-20/01.08.2024). Due to the retrospective nature of the study, the obligation to obtain informed consent was waived by the Ethics Committee.

### Funding

We have no funding relationship relevant to the contents of this paper to disclose.

### Disclosure statement

The authors declare no conflict of interest.

### Data availability statement

The data associated with the paper are not publicly available but are available from the corresponding author on reasonable request.

### Authors’ contributions

Conception and design, administrative support, provision of study materials or patients, collection and assembly of data, data analysis and interpretation, manuscript writing, final approval of manuscript: All authors.

## References

[1] Gursoy S Yazgan S Ucvet A Postpneumonectomy bronchopleural fistula in non-small cell lung cancer patients: incidence, survival, mortality, and treatment analysis. Surg Today 2018; 48: 695–702. 29516277 10.1007/s00595-018-1648-5

[2] Berry MF Harpole DH. Bronchopleural fistula after pneumonectomy. In: Sugarbaker DJ, Bueno R, Colson YL, Jaklitsch MT, Krasna MJ, Mentzer SJ, Williams M, Adams A, editors. Adult chest surgery. 2nd ed. New York: McGraw-Hill Education; 2015;82:683.

[3] Sirbu H Busch T Aleksic I Bronchopleural fistula in the surgery of non-small cell lung cancer: incidence, risk factors, and management. Ann Thorac Cardiovasc Surg 2001; 7: 330–6. 11888471

[4] Andreetti C Menna C D’Andrilli A Multimodal treatment for post-pneumonectomy bronchopleural fistula associated with empyema. Ann Thorac Surg 2018; 106: e337–9. 30009802 10.1016/j.athoracsur.2018.05.094

[5] Pforr A Pages PB Baste JM A predictive score for bronchopleural fistula established using the French database EPITHOR. Ann Thorac Surg 2016; 101: 287–93. 26303974 10.1016/j.athoracsur.2015.06.026

[6] Mammana M Marulli G Zuin A Postpneumonectomy bronchopleural fistula: analysis of risk factors and the role of bronchial stump coverage. Surg Today 2020; 50: 114–22. 31493198 10.1007/s00595-019-01871-0

[7] Skrzypczak PJ Kasprzyk M Piwkowski C. The review of the management and prevention methods of bronchopleural fistula in thoracic surgery. J Thorac Dis 2023; 15: 5268–71. 37969259 10.21037/jtd-23-1231 PMC10636441

[8] Boudaya MS Smadhi H Zribi H Conservative management of postoperative bronchopleural fistulas. J Thorac Cardiovasc Surg 2013; 146: 575–9. 23810114 10.1016/j.jtcvs.2013.04.023

[9] Stratakos G Zuccatosta L Porfyridis I Silver nitrate through flexible bronchoscope in the treatment of bronchopleural fistulae. J Thorac Cardiovasc Surg 2009; 138: 603–7. 19698843 10.1016/j.jtcvs.2008.10.054

[10] Zanotti G Mitchell JD. Bronchopleural fistula and empyema after anatomic lung resection. Thorac Surg Clin 2015; 25: 421–7. 26515942 10.1016/j.thorsurg.2015.07.006

[11] Brunelli A Charloux A Bolliger CT ERS/ESTS clinical guidelines on fitness for radical therapy in lung cancer patients (surgery and chemo-radiotherapy). Eur Respir J 2009; 34: 17–41. 19567600 10.1183/09031936.00184308

[12] Okuda M Go T Yokomise H. Risk factor of bronchopleural fistula after general thoracic surgery: Review article. Gen Thorac Cardiovasc Surg 2017; 65: 679–85. 29027099 10.1007/s11748-017-0846-1

[13] Mazzella A Bertolaccini L Sedda G Pneumonectomy and broncho-pleural fistula: predicting factors and stratification of the risk. Updates Surg 2022; 74: 1471–8. 35416586 10.1007/s13304-022-01290-w

[14] Tokunaga Y Kita Y Okamoto T. Analysis of risk factors for bronchopleural fistula after surgical treatment of lung cancer. Ann Thorac Cardiovasc Surg 2020; 26: 311–9. 32224595 10.5761/atcs.oa.20-00010 PMC7801181

[15] Choi J Oh JY Lee YS Clinical efficacy of adjuvant chemotherapy in stage IB (<4 cm) non-small cell lung cancer patients with high-risk factors. Korean J Intern Med 2022; 37: 127–36. 32872735 10.3904/kjim.2020.011 PMC8747921

[16] Skrzypczak P Roszak M Kasprzyk M The technique of stump closure has no impact on post-pneumonectomy bronchopleural fistula in the non-small cell lung cancer-a cross-sectional study. J Thorac Dis 2022; 14: 3343–51. 36245618 10.21037/jtd-22-240 PMC9562551

[17] Simeone AA. Empyema and bronchopleural fistula following lung resection. Curr Respir Med Rev 2012; 8: 274–9.

[18] Clark JM Cooke DT Brown LM. Management of complications after lung resection: prolonged air leak and bronchopleural fistula. Thorac Surg Clin 2020; 30: 347–58. 32593367 10.1016/j.thorsurg.2020.04.008 PMC10846534

[19] Varoli F Roviaro G Grignani F Endoscopic treatment of bronchopleural fistulas. Ann Thorac Surg 1998; 65: 807–9. 9527218 10.1016/s0003-4975(97)01427-6

[20] Misthos P Kakaris S Sepsas E Surgical management of late postpneumonectomy bronchopleural fistula: the transsternal, transpericardial route. Respiration 2006; 73: 525–8. 16775414 10.1159/000093370

[21] Taghavi S Marta GM Lang G Bronchial stump coverage with a pedicled pericardial flap: an effective method for prevention of postpneumonectomy bronchopleural fistula. Ann Thorac Surg 2005; 79: 284–8. 15620959 10.1016/j.athoracsur.2004.06.108

[22] Musani AI Dutau H. Management of alveolar-pleural fistula a complex medical and surgical problem. Chest 2015; 147: 590–2. 25732439 10.1378/chest.14-2202

[23] Chawla RK Madan A Bhardwaj PK Bronchoscopic management of bronchopleural fistula with intrabronchial instillation of glue (N-butyl cyanoacrylate). Lung India 2012; 29: 11–4. 22345907 10.4103/0970-2113.92350 PMC3276025

[24] Han X Yin M Li L Customized airway stenting for bronchopleural fistula after pulmonary resection by interventional technique: single-center study of 148 consecutive patients. Surg Endosc 2018; 32: 4116–24. 29603006 10.1007/s00464-018-6152-x

[25] Cardillo G Carbone L Carleo F The Rationale for treatment of postresectional bronchopleural fistula: analysis of 52 patients. Ann Thorac Surg 2015; 100: 251–7. 26024752 10.1016/j.athoracsur.2015.03.014

[26] Stamenovic D. Bronchoscopic management of bronchopleural fistula. Curr Thorac Surg 2016; 1: 38–43.

[27] Miller JI Mansour KA Nahai F Single-stage complete muscle flap closure of the postpneumonectomy empyema space: a new method and possible solution to a disturbing complication. Ann Thorac Surg 1984; 38: 227–31. 6236761 10.1016/s0003-4975(10)62243-6

[28] Grunenwald D Assouad J Masmoudi H Preventive bronchial omentoplasty in high risk pulmonary resection following thoracic radiotherapy. J Thorac Oncol 2007; 2: 3–221.17410002

[29] Li W Liu K Liao X Treatment of late left bronchopleural fistula after left pneumonectomy through right thoracic approach assisted by extracorporeal membrane oxygenation. J Cardiothorac Surg 2024; 19: 308. 38822419 10.1186/s13019-024-02805-9 PMC11141060

[30] Moreno P Lang G Taghavi S Right-sided approach for management of left-main-bronchial stump problems. Eur J Cardiothorac Surg 2011; 40: 926–30. 21388823 10.1016/j.ejcts.2010.10.044

[31] Zhu M Yang Y Shi Y A treatment protocol for chronic post-pneumonectomy empyema associated with bronchopleural fistula: a single-centre retrospective study. Int Wound J 2023; 20: 725–31. 36787267 10.1111/iwj.13915 PMC9927892

[32] Horrigan TP Snow NJ. Thoracoplasty: Current application to the infected pleural space. Ann Thorac Surg 1990; 50: 695–9. 2241326 10.1016/0003-4975(90)90664-r

[33] Hubaut JJ Baron O Al Habash O Closure of the bronchial stump by manual suture and incidence of bronchopleural fistula in a series of 209 pneumonectomies for lung cancer. Eur J Cardiothorac Surg 1999; 16: 418–23. 10571088 10.1016/s1010-7940(99)00290-0

